# Student and faculty perceptions: appropriate consequences of lapses in academic integrity in health sciences education

**DOI:** 10.1186/s12909-019-1645-4

**Published:** 2019-06-13

**Authors:** Tina Antill Keener, Marina Galvez Peralta, Melinda Smith, Lauren Swager, James Ingles, Sijin Wen, Mariette Barbier

**Affiliations:** 1 Department of Family and Community Health, School of Nursing, West Virginia University, Morgantown, WV 26506 USA; 2 Department of Pharmaceutical Sciences, School of Pharmacy, West Virginia University, Morgantown, WV 26506 USA; 3 Department of Radiology, West Virginia University School of Medicine, Morgantown, WV 26506 USA; 4 Department of Behavioral Medicine and Psychiatry, West Virginia University School of Medicine, Morgantown, WV 26506 USA; 5 Department of Biostatistics, West Virginia University School of Public Health, Morgantown, WV 26506 USA; 6 Department of Microbiology, Immunology and Cell Biology, West Virginia School of Medicine, West Virginia University, One Medical Center Drive, Morgantown, WV 26506 USA

**Keywords:** Academic integrity, Healthcare education, Clinical settings, Appropriate consequences, Medicine, Nursing, Pharmacy, Dentistry, Sanctions, Perception

## Abstract

**Background:**

A breadth of evidence supports that academic dishonesty is prevalent among higher education students, including students in health sciences educational programs. Research suggest individuals who engage in academic dishonesty may continue to exhibit unethical behaviors in professional practice. Thus, it is imperative to appropriately address lapses in academic dishonesty among health sciences students to ensure the future safety of patients. However, students and faculty have varying perceptions of what constitutes academic dishonesty and the seriousness of breaches in academic dishonesty. The purpose of this study is to gain health sciences faculty and students’ perceptions on the appropriate consequences of lapses in academic integrity.

**Methods:**

Faculty and students from different health care disciplines were asked to complete the anonymous survey in which 10 different academic (non-clinical) and clinical scenarios were presented. For each scenario, students or faculty needed to address their concern and assign an academic consequence that they considered appropriate (ranked from no consequence to dismissal). A mixed-effects model was used to assess the difference of questionnaire scores between subgroups. The study was completed in the Spring of 2017.

**Results:**

A total of 185 faculty and 295 students completed the electronic survey. Across all survey questions (clinical and non-clinical), the perceived severity of the behavior predicted the consequence chosen by the respondent, indicating that both faculty and students assigned what they felt to be appropriate consequences directly based on their values and perceptions. Both faculty and students show congruence in their opinions regarding the perceived seriousness of clinical cases (*p* = 0.220) and the recommended consequences assigned to such lapses (*p* = 0.110). However, faculty and students statistically significantly disagreed in their perception of the severity of non-clinical academic dishonesty scenarios and recommended consequences (*p* < 0.001).

**Conclusions:**

Our research supports the need for collaborative work between faculty and students in putting forth clear guidelines on how to manage and uphold rules related to lapses in academic integrity not only for non-clinical situations, but especially for clinical ones in a health care setting. Recommendations from this research include using an honor code utilized in clinical settings.

## Background

Integrity is defined as having good moral character, upholding moral and ethical codes, and maintaining honesty [1]. The International Center for Academic Integrity (ICAI) asserts that academic integrity is fundamental in education and is the foundation in preparing students to be successful, both personally and professionally [2]. Unfortunately, a breadth of evidence supports breaches of academic integrity are prevalent among higher education students. Recent studies conducted with undergraduate students self-reported ranges from 51 to 78% of students engaged in some type of academic dishonesty [3–6]. Some of the reasons for lapses in academic dishonesty are: pressure to maintain grades, emphasis of grades over comprehension, peer pressure, larger classroom settings [3], reduced fear of getting caught [3], poorer self-control and higher self-oriented thoughts [7], small consequences attached to getting caught, time commitment between work and school [8], and overall actions and attitudes of faculty regarding cheating [7, 9]. Students have reported that some behaviors become acceptable after repeated exposure of cheating that were not detected or lacked a consequence [3, 9, 10]. These findings support identifying and issuing a proper sanction are an important element of curbing or preventing cheating among university students.

Boehm et al. conducted a multi-site, mixed method study aimed at identifying best practices in improving academic integrity and reducing dishonesty. The authors report the best method to reduce academic dishonesty is through faculty training and support [11]. Suggested methods to prevent cheating in the classroom [11] include banning cell phones and other smart devices from testing area [8], having students sign an honor code prior to testing [12, 13], and developing multiple forms of tests. Changing the culture and overall environment is found to curb cheating. Students are less likely to cheat if students perceive faculty as fair and trustworthy [12]. Promoting positive learning environments [14], fostering institutional integrity [15], developing strong faculty-student rapport [16], and engaging students in thoughtful discussions about academic expectations and values [17, 18] are recommended to enhance academic honesty .

All US Health Sciences Academic Certification Boards (Medicine, Pharmacy, Dentistry, Nursing) mandate programs, faculty, and students to uphold professional values and ethics [19–22]. Yet, Health Sciences disciplines are not immune to academic dishonesty. The prevalence of cheating and lapses in academic integrity among health sciences students correlate with that of other disciplines [23, 24]. Cheating behaviors are extremely alarming for students studying health sciences disciplines. Firstly, high ethical standards are an expectation in health sciences students. They play vital roles in society and it is imperative their actions are trustworthy. Secondly, lack of integrity during their education results in a blurred assessment of knowledge, skills and competence. This lack of knowledge could greatly impact the health and safety of future patients and communities at large. Research suggest individuals who engage in academic dishonesty may continue to exhibit unethical behaviors in professional practice [13]. Thus, it is imperative to address appropriately lapses in academic dishonesty among health sciences students to ensure the future safety of patients.

Typically, faculty are first line responders to lapses in academic integrity. Unfortunately, little evidence exist to guide faculty when making difficult decisions on the appropriate consequences of these lapses [10]. Faculty often learn through trial and error of how to best handle difficult situations of students’ lapses of academic integrity with the goal to avoid recurrent problems. University’s academic standards and policies often provide faculty a range of consequences to deal with behaviors of cheating, plagiarism, and unprofessional behaviors. The standards often include options of verbal or written warning and/or reprimand, failure of assignment, failure of course, dismissal from program, professional probation, or expulsion from the University. Due to the lack of clear delineations of what constitute academic dishonesty, faculty often struggle with assigning a fair, congruent, and consistent consequence that aligns with the severity of each situation. Sattar et al. [25] report a significant difference between the recommended consequences for lapses in professional behavior between faculty and students affiliated with medical centers. Compared to faculty members, students affiliated with medical centers were much more likely to recommend more lenient consequences to unprofessional behaviors. Roff et al. reports accord between faculty and students views of appropriate sanctions for first-time offenders of lapses in professionalism among healthcare students. In this study, only four out of 41 offenses included in the survey did students recommend much lower sanctions [26]. Conflicting evidence exists as to the most appropriate sanctions for lapses in academic integrity among health sciences students.

This research was prompted by health sciences faculty who have detected lapses in academic integrity, assigned consequences, and encountered questions from students, colleagues or administrators regarding the congruence between breaches of academic integrity and assigned consequence. Very little evidence exists regarding health sciences faculty and students’ perceptions of which is the appropriate consequences in lapses of academic integrity [25, 26]. To the authors’ knowledge, this evidence has not been collected from a United States sample or within an inter-disciplinary population of health sciences student and faculty. The purpose of this study is to gain health sciences faculty and students’ perceptions on the appropriate consequences of lapses in academic integrity. This study aims to provide insights into the differences between classroom and clinical academic misconduct and provide guidance to those that may need to assign consequences to lapses in academic integrity. The findings from this work may serve as a resource to academicians and administrators in developing and upholding academic standards and protocols within health sciences centers.

## Methods

### Subjects

The participants in this study were undergraduate, graduate, and professional students, medical and pharmacy residents, and faculty enrolled or working within the West Virginia University Health Sciences Center (WVU-HSC). The following schools were included in the study: School of Medicine (907 faculty and 1579 students), School of Pharmacy (45 faculty, and 371 students), School of Nursing (42 faculty and 695 students), and School of Dentistry (71 faculty and 323 students).

### Survey and data collection

A survey including clinical and non-clinical scenarios (Table 1) was designed and distributed to faculty and student populations at West Virginia University Health Sciences Center (WVU-HSC). The study was completed in the Spring of 2017. Program administrators of each school disseminated the electronic survey to all participants. We estimate that the link to the survey was distributed to approximately 3000 undergraduate, professional, and graduate students, and over 1000 faculty and medical and pharmacy residents. Data were collected anonymously through an electronic form during Spring of 2017. The questionnaire contained 10 demographic questions specific to faculty, residents or students: gender, age, school affiliation, years of teaching experience (faculty only), title position (faculty only), education level (students only), first generation college student (students only), prior course in ethics (students only), and prior encounter with academic dishonesty (faculty and students). The demographic survey was then followed by a series of 11 questions including examples of academic and clinical misconduct (Table 1). The opinion of the participants on the severity of each situation was recorded in four categories ranked from “not serious” to “extremely serious”. The participants were then asked to indicate which consequence they thought was most appropriate, with the following options available: “No consequence”, “Failure of the assignment”, “Lower final grade by at least one letter”, “Failure of the course/rotation”, “Placed on academic probation”, “Placed on academic suspension form the program”, “Dismissal from the program”, “Expulsion from the university”, and “No opinion”. The options selected for this answer were based on the West Virginia University guidelines of suggested consequences for academic dishonesty (West Virginia University Board of Governors 15; Student Academic Rights) [27].

**Table 1:** Survey questions

		Scenario
1	Non-clinical	Taking pictures of test material and distributing them online for others to access
2		Paraphrasing material in a written assignment without giving credit to or referencing the original author
3		Copying or sharing answers with another student during the test
4		Sharing password information for an online exam or course so that an unauthorized user can access it
5		Receiving detailed test questions from students who have previously taken the test
6		Making up an excuse or illness to postpone a test for the purpose of allowing more time to study
7	Clinical	Recording patient information in the patient medical record (i.e. vital signs, treatments given, education, or physical examination) as “performed” when it actually was not performed or inaccurately obtained
8		Being involved in or witnessing an adverse patient safety event (i.e. patient fall, improper technique, treatment error) and not reporting it or documenting it appropriately
9		Copying the text from a previous patient assessment and pasting it directly into the patient medical record in a subsequent encounter, when not all aspects of the initial assessment were repeated or verified
10		Posting a de-identified portion of a patient’s medical record or pictures obtained in the clinical care setting on your personal social media account
11		Posting de-identified descriptions of patient care experiences on your social media account

### Data analysis

Only the answers from participants over the age of 18 and affiliated with West Virginia University were used for analysis. Demographic and characteristic data were summarized using descriptive statistics, including means with standard errors or medians with ranges for continuous variables, and frequencies or percentages with contingency tables for categorical variables. For the correlative analysis, Chi-square test and Cochran-Mantel-Haenszel test were used in the data analysis between categorical variables, while Student’s t-test was used in the data analysis for continuous variables. A mixed-effects model was used to assess the difference of questionnaire scores between subgroups in which subjects were regarded as a random factor since there was a correlation between questions from the same subjects.

To facilitate analysis, the consequences of each behavior were scored as follows: “Mild” (sum of answers for “Failure of the assignment” and “Lower final grade by at least one letter”), “Moderate” (sum of the answers for “Failure of the course/rotation”, “Placed on academic probation”, and “Placed on academic suspension from the program”, and “Severe” (sum of answers for “Dismissal from the program” and “Expulsion from the university”). All *p*-values were derived from two-sided tests, and p-value < 0.05 was considered to be statistically significant. Statistical analysis were carried out using SAS 9.1 (SAS Institute, NC) and S-PLUS version 7.0 (Insightful Corp., Seattle, WA) software.

The research performed in this manuscript was approved by the West Virginia University Institutional Review Committee (Protocol #WVU IRB 1607176862). This protocol was approved under the “Exempt” category due to its anonymity.

## Results

### Demographic and survey data

**Fig. 1:**
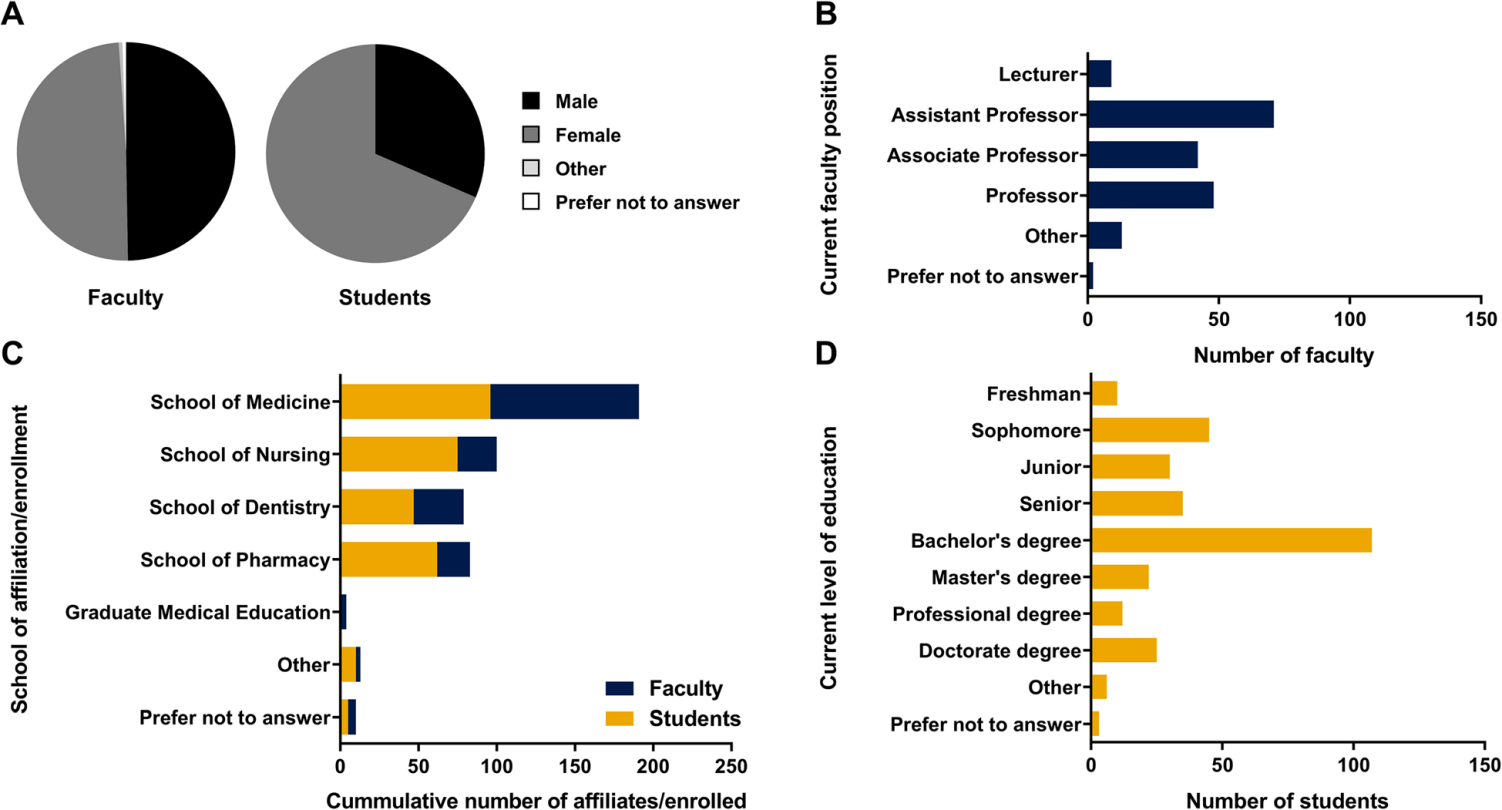
Demographics of the WVU-HSC population who answered the survey. Overall distribution of gender (**a**), current faculty position (**b**), school affiliation or enrollment for both faculty and students (**c**) and current student education level (**d**)

A total of 185 faculty and 295 students completed the anonymous electronic survey, which corresponds to 11.8% overall participation rate. Within the population that answered the survey, there was an equal gender distribution of faculty (male and female) but a predominance of female students (31.5% males and 68.5% females, *p* < 0.001) (Fig. 1a). This disparity in the student population is likely related to a gender mismatch in the number of students that are enrolled in nursing and dental hygiene degree programs at our institution. Additional demographic data collected during the survey included current faculty appointment rank (Fig. 1b), school affiliation or enrollment (Fig. 1c), and student’s education level prior to enrolment in their current program (Fig. 1d).

We observed that the average age of the faculty was 50.5 ± 12.8 (*n* = 166), with an average teaching experience of 17.4 ± 11.7 years (*n* = 174). The average age reported by the student population was 25.0 ± 6.0 (*n* = 273), and a large proportion of the students surveyed had already obtained a degree and were enrolled with the School of Medicine. These data reflect the overall student and faculty demographics of the WVU-HSC.

#### Perception and consequences responses from students and faculty

**Fig. 2:**
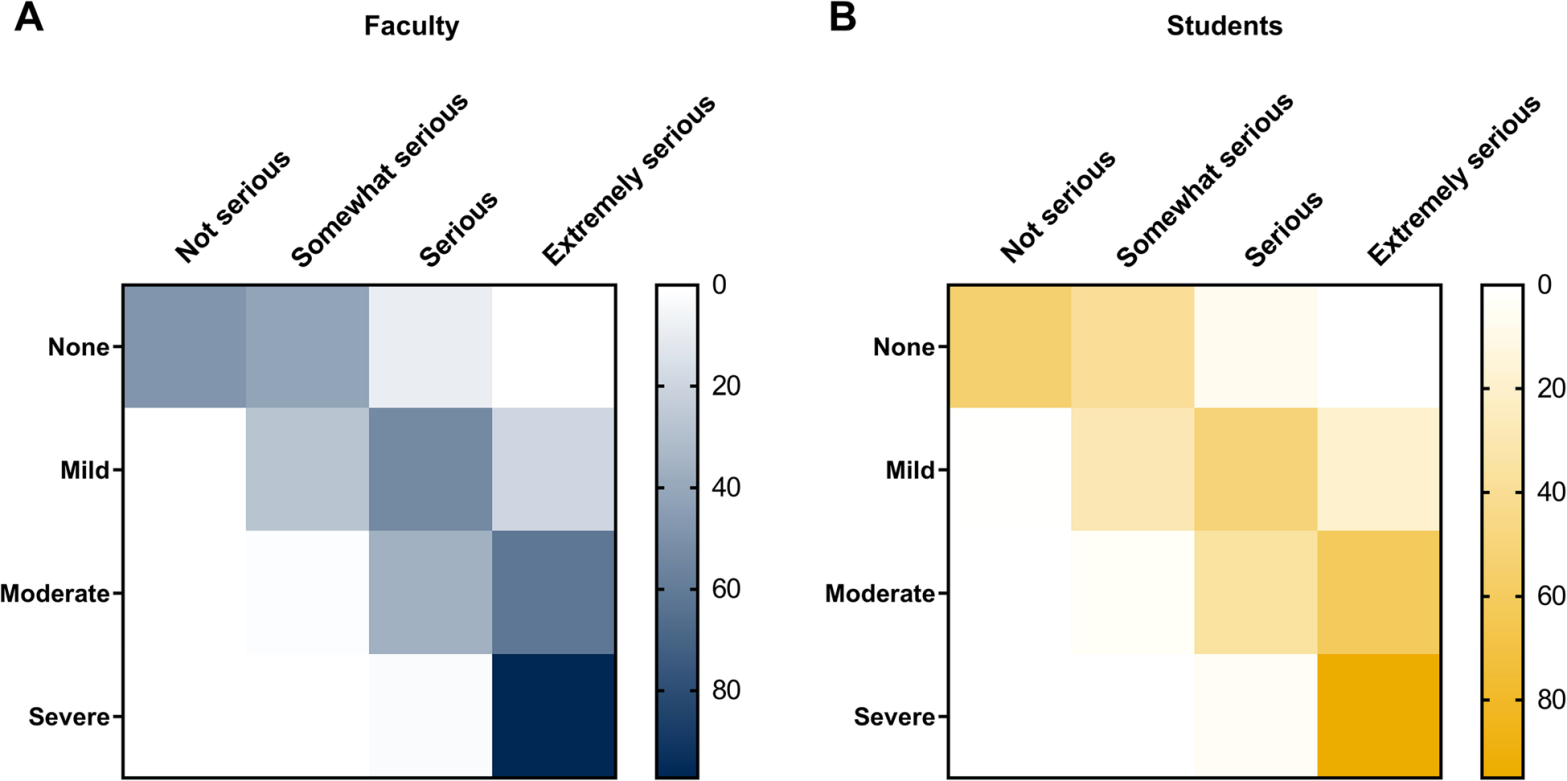
Distribution of perception and consequence of each scenario. Heat map representation of the association between perception and consequence for all scenarios for faculty (**a**) and students (**b**). Values represent the average number of responses for each group across all 11 scenarios

Across all survey questions (clinical and non-clinical), the perceived severity of the behavior predicted the consequence chosen by the respondent (Fig. 2), indicating that both faculty and students assigned what they felt to be appropriate consequences directly based on their values and perceptions. If an event was perceived as not serious, no consequence was felt to be appropriate. If the consequence was somewhat serious, a mild consequence (i.e. failure of an assignment or lowered final grade) was recommended. For serious or extremely serious events, severe or extremely severe consequences (i.e. dismissal from the program or expulsion from the university) were recommended by the survey respondents regardless of their faculty or student designation (Fig. 2).

**Fig. 3:**
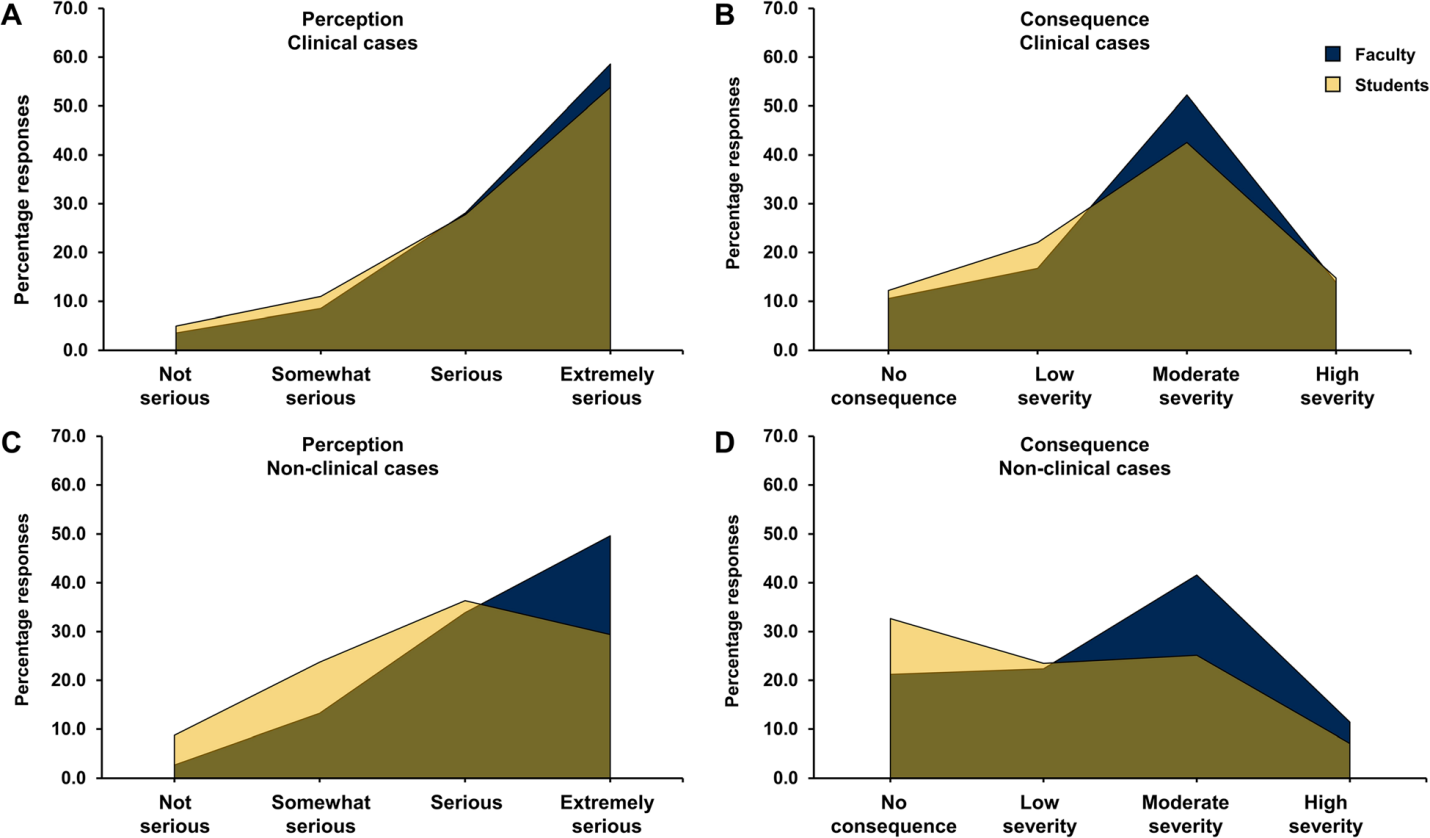
Distribution of overall perception and consequence of clinical and non-clinical scenarios. Percentage of faculty (blue) or students (gold) categorizing the seriousness of How are clinical (**a**) and non-clinical (**c**) cases of academic misconduct. Attributed severity of the consequence of clinical (**b**) and non-clinical (**d**) cases of academic dishonesty

Overall, both faculty and students show significant congruence in their opinions regarding the perceived seriousness of clinical cases (*p* = 0.220) and the recommended consequences assigned to such lapses (*p* = 0.110) (Fig. 3a and b) For example, if a faculty or a student considered that the case was not serious, a less severe consequence would be assigned, while if the case was perceived as serious, a severe consequence was attributed. However, faculty and students statistically significantly disagreed in their perception of the severity of non-clinical academic dishonesty scenarios (*p* < 0.001), leading to noted differences in recommended consequences for academic/non-clinical scenarios (*p* < 0.001) (Fig. 3c and d).

#### Variables that impacted perception and consequences

**Fig. 4:**
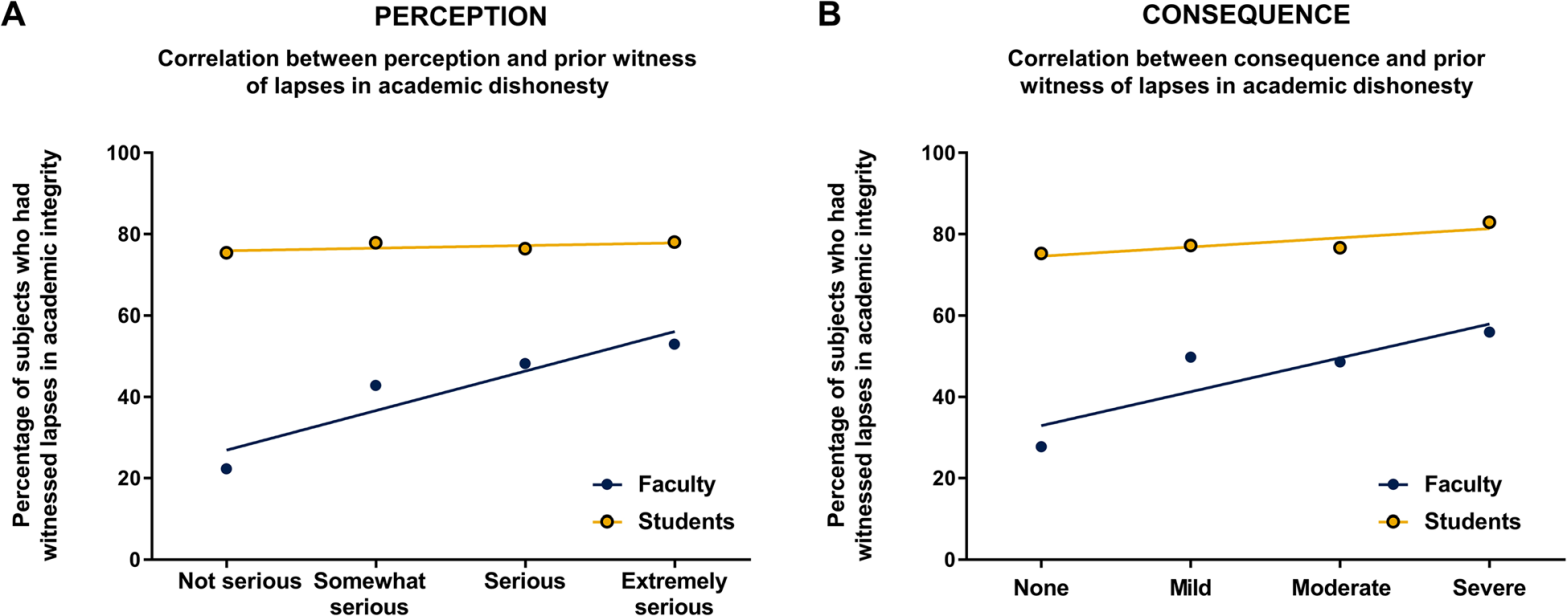
Association between prior exposure to lapses in academic integrity, perception, and consequence. Linear correlation curves for perception (**a**) and consequence (**b**) for faculty (blue) and students (gold). Values represent the average number of responses for each group across all 11 scenarios

When studying the role of prior encounters of students and faculty on their perception of lapses in academic dishonesty and associated consequences, we observed that a larger proportion of the students had experienced situations of academic misconduct than faculty (Fig. 4a and b). Overall, faculty that had previously witnessed lapses in integrity were much more likely to perceive events as more serious (*p* = 0.004), and more likely to associate a more severe consequence to those behaviors, although the correlation was not significant (*p* = 0.090). However, the perception (*p* = 0.170) and consequence (*p* = 0.850) associated to each case by the student was not significantly affected by whether they had witnessed prior lapses in academic dishonesty in the past or not (Fig. 4). There were also some differences explained by the educational level of the students. Student who have not received a bachelor’s degree or higher were more likely to perceive clinical scenarios as more severe (*p* = 0.022). On the other hand, students with a bachelor’s degree or higher perceived non-clinical scenarios more seriously (*p* = 0.027) and attributed a higher consequence (*p* < 0.001).

Several parameters did not influence perception or consequences related to academic integrity lapses. These parameters were the faculty work experience (*p* = 0.190 for concern and *p* = 0.120 for consequence), and students previously taking an ethics course (*p* = 0.570 for concern and *p* = 0.290 for consequence).

#### Consequences for academic misconduct

**Fig. 5:**
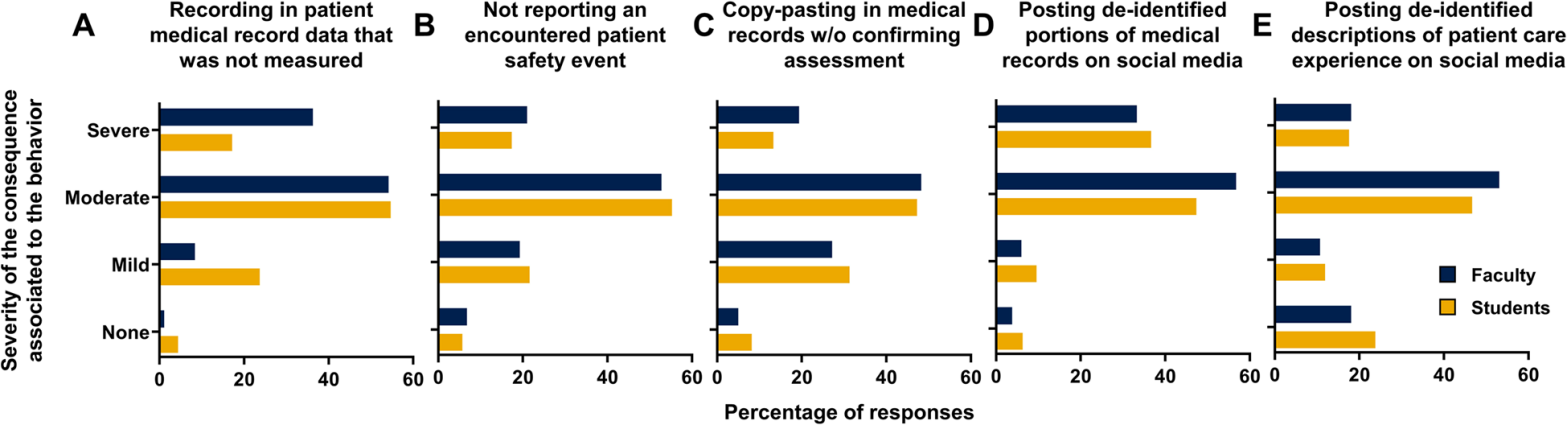
Faculty and student consequences for clinical scenarios. Percentage of the responses recorded for faculty (blue) or students (gold) for each clinical scenario (**a**-**e**) for the consequence associated to each scenario

Faculty and students aligned perceptions of seriousness in clinical cases led to significant congruence in their opinions regarding the appropriate consequences for clinical lapses (Fig. 5). Questions that were perceived as moderately serious such as patient’s safety events, cutting and pasting into the electronic medical record, and posting de-identified written descriptions of patient experiences on social media were felt to represent the need for moderate consequences (i.e. failure of a course, paced on probation or suspension). Also, recording data into the medical record that were not actually collected, or posting photographic material even if the identification of the patient was omitted, was viewed as a more moderate or severe offense, and more moderate to severe consequences were recommended.

Divergence of perceptions in seriousness of non-clinical cases between faculty and students led to continued divergence in consequences for non-clinical scenarios that these findings were not statistically significant making it difficult to make recommendations about non-clinical consequences and highlighting need for further research and collaboration.

## Discussion

The results of this survey study hold promise for health sciences campuses in establishing and refining policies and consequences around academic integrity lapses in clinical situations. Non-clinical scenarios will be discussed later on a future study. While there was less agreement between students and faculty regarding the nature of nonclinical lapses, there was clearly a trend that a consequence was needed for any perceived lapse. For non-clinical cases, there were very few respondents that felt “no consequence” was an option even for minor offenses.

Overall respondents wanted to see appropriate consequences that matched the perception of the severity of the offence. This goes against previous literature reporting students did not feel consequences were warranted in several examples of lapses of academic integrity and professionalism and suggested some behaviors be ignored [25]. Both students and faculty showed significant agreement on the need to address lapses in clinical behaviors viewed to be as unethical. The clinical lapses may relate to deficits in professionalism that hold stronger meaning for survey respondents because when an actual patient or healthcare experiences are involved the level of risk feels more imminent and relatable.

Our research supports the need for collaborative work between faculty and students in putting forth clear guidelines on how to manage and uphold rules related to lapses in academic integrity not only for non-clinical situations [11], but for clinical ones in a health care setting. This underpins the importance of faculty and students working together to create a supportive, positive learning environment with a zero tolerance for academic dishonesty. Proactive factors may better promote academic honesty than punitive sanctions [11]. Robinson & Glazer recommend shifting motivating factors of academic honesty from fear of getting caught to a love of learning [14]. In this study, both students and faculty perceived lapses of academic integrity in clinical practice as more serious than lapses of academic integrity in the classroom or other non-clinical related learning environments. Thus, the results of this study may be helpful for faculty in how they communicate academic integrity with students. Faculty should foster intrinsic motivation to maintain academic integrity among their students through creating a learning environment that reinforces the importance of meaningful connections between knowledge gained in non-clinical settings and application to safe, high-quality clinical practice [14].

This research could be used as a guideline for health sciences centers that wish to explore establishing more clear and meaningful programs to address lapses in academic integrity. A recent study found students who are more tolerant of cheating and involved in classroom dishonesty were more likely to participate in dishonest clinical behaviors [28]. Thus, it is essential to address and assign appropriate sanctions related to academic dishonesty in both classroom and clinical settings. With nearly 80% of students witnessing lapses in academic integrity, there is significant underreporting occurring within academic systems that also needs addressed.

One option may be a consideration of an honor code that could be utilized in non-clinical and clinical settings in health sciences campuses. To the research team’s knowledge, there is no evidence of an honor code utilized in clinical settings. As professionalism on health sciences campuses is highly regarded, an honor code may reinforce the magnitude of ethical focus if utilized for each rotation and service within the clinical rotation curriculum of its students. General conversations regarding academic honesty is found to have little effect. Academic honesty is more favorable when students are provided clear and specific expectations of behaviors related to each assignment [18], or in this instance, each clinical rotation or service.

Another option beyond honor codes may be the creation of a health sciences academic integrity committee comprised of faculty and students with a board range of backgrounds and perspectives on academic integrity to which individual cases of violations integrity are assigned. Evidence support initiatives aimed at involving students in developing academic honesty policies and assigning sanctions in lapses of academic integrity have reduced cheating [11]. The committee can act as the investigators regarding the facts in the case and serve in mediation of what an appropriate consequence should be, taking into account this survey’s data as well as providing a comprehensive look at the seriousness of the facts. This may lend universities more perspective in how to proceed with outcomes rather than relying on decisions based on only one faculty member’s or one administrator’s viewpoint, finding ways to increase report, or educate students on expectations.

Despite the need to move forward in managing academic lapses in integrity in Health Sciences Campuses, this survey data still hold several limitations. Completing a survey regarding how someone perceives they will respond does not correlate with how individuals will actually respond when real people or real situations are at hand. The other challenge to this survey is that by the use of short narrative stems without much detail we did not account for how different disciplines with in health sciences may perceive the larger context of a narrative. This study is unable to interpret differences and how one discipline of healthcare may have responded compared to another as there might be more interpretation in the brief stems than this study could explore. Finally as technology continues to evolve ad there is an increasing demand for electronic health records and data collection and ethics regarding electronic media it is likely that these policies will need to continue to be adapted and refined over time as new concerns regarding lapses in academic integrity may emerge.

## Conclusions

Health sciences students are held to high ethical standards. However, prevalence of cheating and lapses in academic integrity among health sciences students correlate with that of other disciplines. This research supports the need for collaborative work among administrators, faculty, students, and stakeholders in preventing academic dishonesty and assigning appropriate consequences to lapses of academic integrity. It is imperative academic integrity in health sciences education be maintained to ensure future health care professionals are adept at providing safe, quality care.

## Data Availability

The dataset s used and/or analyzed during the current study are available from the corresponding author on a reasonable request.

## Abbreviations

ICAI: International Center for Academic Integrity
w/o: without
WV-CTSI: West Virginia Clinical and Translational Science Institute
WVU IRB: West Virginia University Institutional Review Committee
WVU-HSC: West Virginia University Health Sciences Center

## References

[1] Dictionary.com. Integrity. http://www.dictionary.com/browse/integrity. Accessed 14 Oct 2017.

[2] IThe Fundamental Values of Academic Integrity Second Edition International Center for Academic Integrity T. Fishman, ISBN: 978-0-9914906-7-7.

[3] Freiburger TL, Romain DM, Randol BM, Marcum CD (2017). Cheating behaviors among undergraduate college students: results from a factorial survey. J Crim Justice Educ.

[4] Mortaz Hejri S, Zendehdel K, Asghari F, Fotouhi A, Rashidian A (2013). Academic disintegrity among medical students: a randomised response technique study. Med Educ.

[5] Hensley LC, Kirkpatrick KM, Burgoon JM (2013). Relation of gender, course enrollment, and grades to distinct forms of academic dishonesty. Teach High Educ.

[6] Park E-J, Park S, Jang I-S (2013). Academic cheating among nursing students. Nurse Educ Today.

[7] Yu H, Glanzer PL, Sriram R, Johnson BR, Moore B (2017). What contributes to college students’ cheating? A study of individual factors. Ethics Behav.

[8] Best College Reviews. Cheating in college: the numbers and research. https://www.bestcollegereviews.org/cheating/. Accessed 14 Oct 2017.

[9] Beasley EM (2014). Students reported for cheating explain what they think would have stopped them. Ethics Behav.

[10] Stonecypher Karen, Willson Pamela (2014). Academic Policies and Practices to Deter Cheating in Nursing Education. Nursing Education Perspectives.

[11] Boehm P, Justice M, Weeks S. Promoting academic integrity in online education. The community college Enterprise; 2009.

[12] Tatum H, Schwartz BM (2017). Honor codes: evidence based strategies for improving academic integrity. Theory Pract.

[13] LaDuke RD (2013). Academic dishonesty today, unethical practices tomorrow?. J Prof Nurs.

[14] Robinson JA, Glanzer PL. Building a culture of academic integrity: what students perceive and need. Coll Stud J 2017;51:209.

[15] Olafson L, Schraw G, Kehrwald N (2014). Academic dishonesty: behaviors, sanctions, and retention of adjudicated college students. J Coll Stud Dev.

[16] Tatum HE, Schwartz BM, Hageman MC, Koretke SL (2018). College students’ perceptions of and responses to academic dishonesty: an investigation of type of honor code, institution size, and student–faculty ratio. Ethics Behav.

[17] Oran NT, Can HÖ, Şenol S, Hadımlı AP (2016). Academic dishonesty among health science school students. Nurs Ethics.

[18] Broeckelman-Post MA (2008). Faculty and student classroom influences on academic dishonesty. IEEE Trans Educ.

[19] Accreditation council for pharmacy education. Accreditation standards and key elements for the professional program in pharmacy leading to the doctor of pharmacy degree. 2015. https://www.acpe-accredit.org/pdf/Standards2016FINAL.pdf. Accessed 24 Feb 2019.

[20] ACGME Common Program Requirements. https://www.acgme.org/Portals/0/PFAssets/ProgramRequirements/CPRs_2017-07-01.pdf. Accessed 24 Feb 2019.

[21] Commission on dental accreditation accreditation standards for dental hygiene education programs. www.ada.org/coda. Accessed 24 Feb 2019.

[22] The Essentials of Baccalaureate Education for Professional Nursing Practice. 2008. http://www.aacnnursing.org/portals/42/publications/baccessentials08.pdf. Accessed 24 Feb 2019.

[23] Wajda-Johnston V (2001). A., Handal PJ, Brawer P a., Fabricatore AN. Academic dishonesty at the graduate level. Ethics Behav.

[24] McCabe DL, Butterfield KD, Treviño LK (2006). Academic dishonesty in graduate business programs: prevalence, causes, and proposed action. Academy of Management Learning and Education.

[25] Sattar K, Roff S, Meo SA (2016). Your professionalism is not my professionalism: congruence and variance in the views of medical students and faculty about professionalism. BMC Med Educ.

[26] Roff S, Chandratilake M, Mcaleer S, Gibson J (2011). Preliminary benchmarking of appropriate sanctions for lapses in undergraduate professionalism in the health professions. Med Teach.

[27] University WV. No Title.

[28] Bultas MW, Schmuke AD, Davis RL, Palmer JL (2017). Crossing the “line”: college students and academic integrity in nursing. Nurse Educ Today.

